# *AfAP2-1*, An Age-Dependent Gene of *Aechmea fasciata*, Responds to Exogenous Ethylene Treatment

**DOI:** 10.3390/ijms17030303

**Published:** 2016-02-27

**Authors:** Ming Lei, Zhi-Ying Li, Jia-Bin Wang, Yun-Liu Fu, Meng-Fei Ao, Li Xu

**Affiliations:** Ministry of Agriculture Key Laboratory of Crop Gene Resources and Germplasm Enhancement in Southern China, Institute of Tropical Crop Genetic Resources, Chinese Academy of Tropical Agricultural Sciences, Danzhou 571737, China; leiming_catas@126.com (M.L.); xllizhiying@vip.163.com (Z.-Y.L.); jiabinwangfuhu@sina.com (J.-B.W.); fyljj_2007@126.com (Y.-L.F.); 17889981612@163.com (M.-F.A.)

**Keywords:** *Aechmea fasciata*, *APETALA2*, transcriptional activator, flowering, bromeliad

## Abstract

The Bromeliaceae family is one of the most morphologically diverse families with a pantropical distribution. To schedule an appropriate flowering time for bromeliads, ethylene is commonly used to initiate flower development in adult plants. However, the mechanism by which ethylene induces flowering in adult bromeliads remains unknown. Here, we identified an *APETALA2* (*AP2*)-like gene, *AfAP2-1*, in *Aechmea fasciata*. AfAP2-1 contains two AP2 domains and is a nuclear-localized protein. It functions as a transcriptional activator, and the activation domain is located in the C-terminal region. The expression level of *AfAP2-1* is higher in juvenile plants than in adult plants, and the *AfAP2-1* transcript level was rapidly and transiently reduced in plants treated with exogenous ethylene. Overexpression of *AfAP2-1* in *Arabidopsis thaliana* results in an extremely delayed flowering phenotype. These results suggested that *AfAP2-1* responds to ethylene and is a putative age-dependent flowering regulator in *A. fasciata*.

## 1. Introduction

The Bromeliaceae family, which consists of 3248 species in 58 genera [1,2], is one of the most morphologically diverse families with a pantropical distribution [3]. Due to the astonishing flavour and fragrance of certain fruits, some species, such as pineapple (*Ananas comosus*), were domesticated in northern South America possibly more than 6000 years ago [4,5]. Except for the edible pineapple, the vast majority of bromeliads cultivated worldwide are appreciated mainly for their ornamental value. Many bromeliads utilize crassulacean acid metabolism (CAM), a photosynthetic carbon fixation pathway that permits them to be highly productive in water-limited environments [5,6]. Thus, bromeliads, such as pineapple, are subtropical and tropical flowering plants with significant economical importance [7].

The onset of flowering is critical during the transition from the vegetative to reproductive phase in the plant lifestyle [8]. To date, five genetic pathways relevant to flowering have been identified in the model species *Arabidopsis thaliana*, namely, the photoperiod, vernalization, gibberellic acid, autonomous and ageing pathways [9]. During this complex developmental process, a subset of flowering-promoter and -suppressor genes participate in multiple pathways, such as *APETALA2* (*AP2*) and five other *AP2*-like transcription factors (TFs), including *TARGET OF EAT 1* (*TOE1*), *TOE2*, *TOE3*, *SCHLAFMÜTZE* (*SMZ*), and *SCHNARCHZAPFEN* (*SNZ*) [10,11,12,13]. microRNA172 (miR172), a small, non-coding RNA that can complement a region of its target genes, can post-transcriptionally repress all members of the AP2 family [14,15,16,17]. *toe1* mutants have previously been shown to flower significantly early, and this effect was enhanced in *toe1toe2* double-mutants [11,14]. Additionally, neither *smz* nor *snz* mutants display any notable early flowering phenotypes, whereas *toe1toe2smzsnz* quadruple mutants were found to flower even earlier than *toe1toe2* double mutants but continued to flower significantly later than plants that constitutively express miR172 [12,14,15]. The hextuple mutant lacking all six *AP2* family genes exhibits even an earlier flowering phenotype than *toe1toe2smzsnz* quadruple mutants, but its phenotype is similar to that of miR172-overproducing plants [18]. The overexpression of all of these genes, except *TOE3*, results in delayed flowering [11,12,14,18]. As floral repressors, the expression of *TOE1*, *TOE2*, *SMZ* and *SNZ* transcripts displays circadian periodicity with a peak in the early morning under long day conditions, repressing the expression of *Flowering Locus T* (*FT*), which is considered to be the florigen, in the early morning and afternoon [13]. Further investigations demonstrated that at least TOE1 and SMZ were able to bind directly to the promoter sites of *FT*, and the repression of *FT* and other flowering integrators is essential to prevent flowering [12,13]. These investigations indicated that all six AP2 family members act redundantly to repress flowering.

As a gaseous hormone, ethylene is essential for regulating variable plant developmental processes, including flowering [19,20,21]. In *Arabidopsis*, the activation of ethylene production can reduce bioactive gibberellin acid (GA) levels by inhibiting the protein kinase CONSTITUTIVE TRIPLE RESPONSE1 (CTR1) and increasing the levels of the downstream TF Ethylene Insensitive 3 (EIN3) [20,22]. Reduced bioactive GA levels can then increase the accumulation of DELLA proteins, a subfamily of GRAS TFs, and delay flowering by inhibiting the upregulation of the floral inducers *LEAFY* (*LFY*) and *SUPPRESSOR OF OVEREXPRESSION OF CONSTANS 1* (*SOC1*) [20]. Interestingly, exogenous ethylene can induce the flowering of Bromeliads, such as *A. comosus* and *Aechmea fasciata*. Aviglycine ((*S*)-*trans*-2-amino-4-(2 aminoethoxy)-3-butenoic acid hydrochloride, AVG), an inhibitor of ethylene biosynthesis, can delay the natural flowering of pineapple (*A. comosus* var. *comosus*) [23,24]. The silencing of one of 1-amino-cyclopropane-1-carboxylate synthase (ACC synthase) genes, *ACACS2*, can also delay flowering in *A. comosus* [25]. These studies demonstrated that exogenous ethylene mediates the flowering of *A. comosus* by stimulating the synthesis of endogenous ethylene. However, the precise mechanism by which ethylene induces flowering in bromeliads remains unknown, posing a large obstacle to shorten the long generation cycle and flowering time and culture new varieties using attractive genetic engineering tools [6,26].

In this study, we provide the first report of the isolation and functional characterization of *AfAP2-1*, an *AP2*-like gene in *A. fasciata*, a popular ornamental flowering bromeliad. Transactivation assays in yeast demonstrated that the activation domain is located in the C-terminal region. Furthermore, the expression of *AfAP2-1* transcripts responded to plant age and ethylene treatment in *A. fasciata* and that the overexpression of *AfAP2-1* in Columbia-0 (Col-0) background *Arabidopsis* (Wild Type, WT) significantly delayed flowering in both short-day (SD) and long-day (LD) conditions. These results all support the conclusion that AfAP2-1, a TF of the AP2 family, may play a pivotal role in regulating flowering time in *Arabidopsis*.

## 2. Results

### 2.1. Isolation and Sequence Analysis of AfAP2-1

AP2-like cDNA was isolated from *A. fasciata* via the rapid-amplification of cDNA ends (RACE) strategy (primers are listed in Table S1) combined with the analysis of transcriptome data. The cDNA of AfAP2-1 (GenBank accession no. KU350628) was 2057 bp long, with a 375-bp 5′ untranslated region (UTR), a 320-bp 3′ UTR and a 1362-bp open reading frame (ORF). A comparison of the AfAP2-1 cDNA with five miR172s in *A. fasciata* revealed a putative miR172 recognition site (1238CTGCAGCATCATCAGGATTCT1258) within the sequence encoding the C terminus of AfAP2-1, which is outside of the conserved AP2 domains.

**Figure 1:**
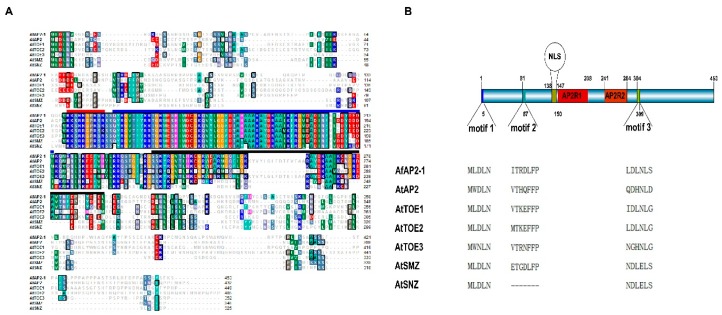
Alignment of the deduced amino acid sequences of AfAP2-1 with AP2 and AP2-like proteins of Arabidopsis and the structure of AfAP2-1. (**A**) Alignment of the deduced amino acid sequences of AfAP2-1 with AP2 and AP2-like proteins of Arabidopsis. The red line indicates the completely conserved putative nuclear localization signal (NLS) sequences, and the blue and black lines indicate the two AP2 domains (AP2R1 and AP2R2). At: Arabidopsis thaliana. AtAP2: AEE86718; AtTOE1: AEC08137; AtTOE2: AED97280; AtTOE3: AED98311; AtSMZ: AEE79323; AtSNZ: AEC09650; (**B**) The structure of AfAP2-1. The sequences of three motifs of AfAP2-1 and all six AP2 members in Arabidopsis are shown. “-“ represents the gap produced in the multiple alignments.

The AfAP2-1 cDNA was predicted to encode a 453-amino acid protein (Figure 1A) with a molecular weight and isoelectric point (pI) of 49.50 kDa and 8.52, respectively. A BLASTP search indicated that the deduced polypeptide is similar to AP2 or AP2-like proteins from a range of plant species (Figure 1A). A sequence comparison between AfAP2-1 and six AP2 family members in Arabidopsis showed that (1) the putative ten-amino-acid localization signal (amino acids from 138 to 147) is completely conserved and (2) the double AP2 domains (amino acids from 150 to 208 and from 241 to 284) and the linker between them are highly similar (Figure 1A,B).

**Figure 2:**
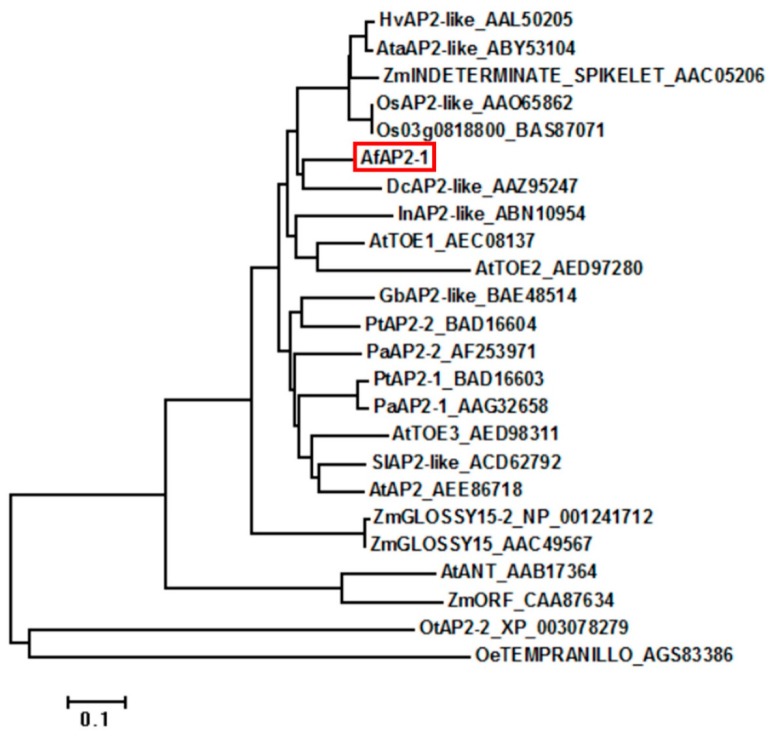
Phylogenetic analysis of AfAP2-1 and AP2 and AP2-like proteins from other plant species. The phylogenetic tree was constructed with MEGA version 6 using the neighbour-joining method with 1000 bootstrap replicates. The scale bar indicates the branch length. Species abbreviations: Af: *Aechmea fasciata*; At: *Arabidopsis thaliana*; Ata: *Aegilops tauschii*; Dc: *Dendrobium crumenatum*; Gb: *Ginkgo biloba*; Hv: *Hordeum vulgare* ssp. vulgare; In: *Ipomoea nil*; Oe: *Olea europaea*; Os: *Oryza sativa* Japonica Group; Ot: *Ostreococcus tauri*; Pa: *Picea abies*; Pt: *Pinus thunbergii*; Sl: *Solanum lycopersicum*; Zm: *Zea mays*.

A phylogenetic analysis of AfAP2-1 and 23 AP2 and AP2-like proteins from other species further confirmed that AfAP2-1 is a member of the AP2 subfamily (Figure 2). The result revealed that AfAP2-1 (indicated with a red box) was more closely related to the AP2-like proteins of monocotyledonous herbaceous plants, such as *Dendrobium crumenatum*, than the AP2-like proteins of woody plants, such as *Olea europaea*.

### 2.2. Transcript Profiling of AfAP2-1 in A. fasciata

**Figure 3:**
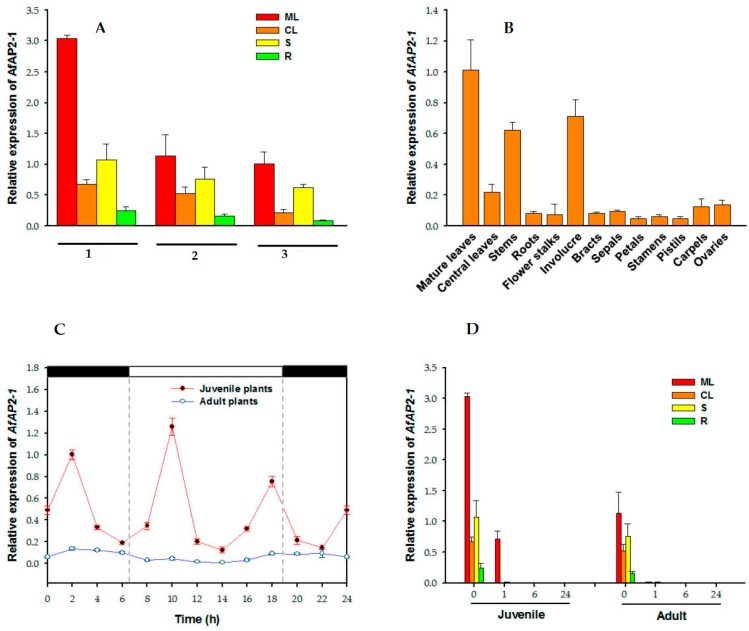
Expression of *AfAP2-1* transcripts in various tissues of *A. fasciata*. (**A**) The expression level of *AfAP2-1* transcripts in various tissues of juvenile and adult plants. 1, juvenile plants; 2, adult plants before differentiation; 3, adult plants of 39-DAF flowering. ML, mature leaves; CL, central leaves; S, stems; R, roots; (**B**) Expression level of *AfAP2-1* transcripts in various tissues of 39-DAF flowering adult plants; (**C**) Expression level of *AfAP2-1* transcripts in central leaves of *A. fasciata* grown in the greenhouse at natural daily light rhythm. Samples were collected at 2 h intervals. The dark bars indicate the dark period, and the white bar indicates the light period; (**D**) The expression level of *AfAP2-1* transcripts in various tissues of *A. fasciata* in response to exogenous ethylene treatment for different times. 0, 1, 6 and 24 indicate the exogenous ethylene treatment time (h). Three independent experiments were performed, and values are shown as means and error bars indicate standard deviation (*n* = 3).

To gain insight into the role of AfAP2-1 in *A. fasciata*, we used quantitative real-time PCR (qRT-PCR) to analyse its transcription levels in various vegetative and reproductive organs of both juvenile and adult plants (Figure 3A,B). *AfAP2-1* transcripts were detected in all vegetative organs, including mature leaves, central leaves, stems and roots. In all developmental stages tested, *AfAP2-1* mRNA was more abundant in mature leaves than in all other organs (Figure 3A,B). In addition, the accumulation of *AfAP2-1* transcripts showed significant changes during development, with relatively high levels in juvenile plants, a decrease in adult plants prior to flower bud differentiation, and a further decrease in 39-day-after-flowering (DAF) adult plants (Figure 3A). Interestingly, the qRT-PCR results showed that the expression of *AfAP2-1* was low in multiple flowering organs, especially in bracts, sepals, petals, stamens and pistils (Figure 3B). These results suggest that *AfAP2-1* may play a role in phase transition rather than in floral meristem identity.

### 2.3. Relative Expression of AfAP2-1 under the Control of the Circadian Rhythm

To investigate the circadian patterns of *AfAP2-1* expression, we examined central leaves of both juvenile and adult plants grown under a natural daily light rhythm in a greenhouse (ambient temperature of 30–32 °C) on 2 August 2014 (Figure 3C). In juvenile plants, the expression of *AfAP2-1* exhibited a peak at 02:00 h. In addition, another peak was observed at 10:00 h, approximately 3.5 h after sunrise. The third peak emerged at 18:00, 1 h before sunset. Furthermore, similar morning and afternoon peaks were also observed in adult plants, although the expression of *AfAP2-1* transcripts was consistently lower than in juvenile plants at every detected time point (Figure 3C). These observations indicated that *AfAP2-1* expression responded to the natural daily light rhythm and was unrelated to plant age.

### 2.4. Response to Exogenous Ethylene Treatment

To investigate the possible involvement of *AfAP2-1* in response to exogenous ethylene treatment, we also evaluated its expression in various organs of juvenile and adult plants exposed to exogenous ethylene treatment (Figure 3D). The *AfAP2-1* transcripts showed a rapid and transit reduction in all examined organs after 1 h of treatment with ethylene. After 6 h, *AfAP2-1* expression could not be detected in any organ in juvenile and adult plants. This result indicated that the transcription of *AfAP2-1* was significantly reduced by exogenous ethylene treatment.

### 2.5. AfAP2-1 Exhibits Transactivation Activity in Yeast

**Figure 4:**
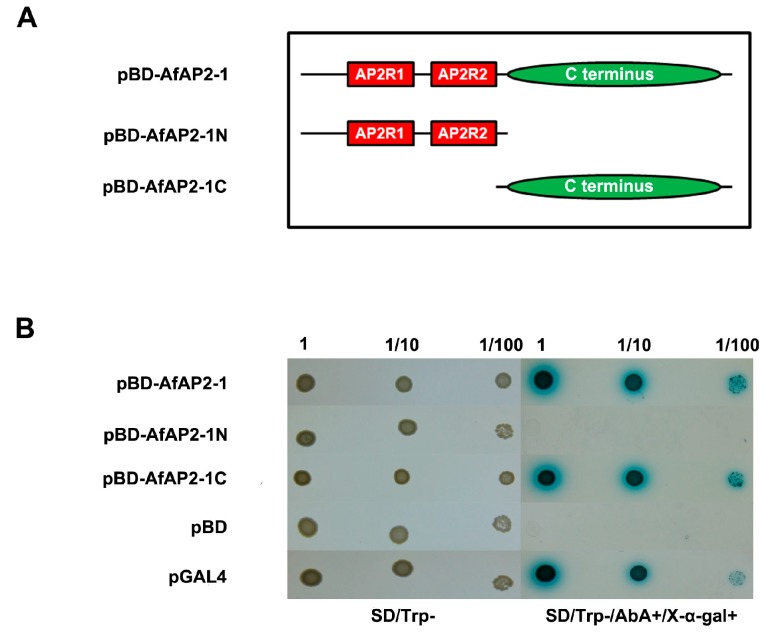
Transactivation activity assay of AfAP2-1 in yeast cells. (**A**) Schematics of the constructs of pGBKT7 (pBD) vectors fused with full-length AfAP2-1 (pBD-AfAP2-1), the N terminus of AfAP2-1 (pBD-AfAP2-1N) and the C terminus of AfAP2-1 (pBD-AfAP2-1C); (**B**) Transactivation activity assay in yeast cells. pBD and pGAL4 plasmids were transformed into Y2HGold cells and used as negative and positive controls, respectively. Yeast clones containing the right constructs grew on SD/Trp- medium at dilutions of 1, 1/10 and 1/100. Three to five days later, the right clones were transferred onto SD/Trp-/AbA+/X-α-gal+ medium for continuous growth for three days to test their transactivation activities. SD: synthetic dropout; AbA: Aureobasidin A; SD/Trp-: SD medium without Trp; SD/Trp-/AbA+/X-α-gal+: SD medium without Trp, but with 40 mg/L X-α-gal and 200 µg/L AbA.

AfAP2-1 contains two AP2 domains in the N terminus, making it a member of the AP2 TF subfamily of the AP2/EREBP superfamily. A transactivation assay was performed in yeast to assess the function of this protein as a transcriptional activator. The sequences encoding the N terminus, C terminus and full-length protein of AfAP2-1 were individually fused with the GAL4 binding domain using the pGBKT7 vector and transformed into the yeast strain Y2HGold, which contains *AUR1-C* and *MEL1* reporter genes (Figure 4A). The expression of *AUR1-C* and *MEL1* can confer strong resistance to the highly toxic drug Aureobasidin A (AbA) and turn the yeast cells blue in the presence of the chromagenic substrate X-α-Gal. Yeast cells carrying pGBKT7-AfAP2-1 and pGBKT7-AfAP2-1C, as well as the positive control, but not pGBKT7-AfAP2-1N, can grow on plates containing synthetic dropout (SD) /Trp-/X-α-Gal+/AbA+ medium, indicating that AfAP2-1 has transactivity and that the transactivation domain is located in the C terminus (Figure 4B).

### 2.6. Subcellular Localization of AfAP2-1 Protein

**Figure 5:**
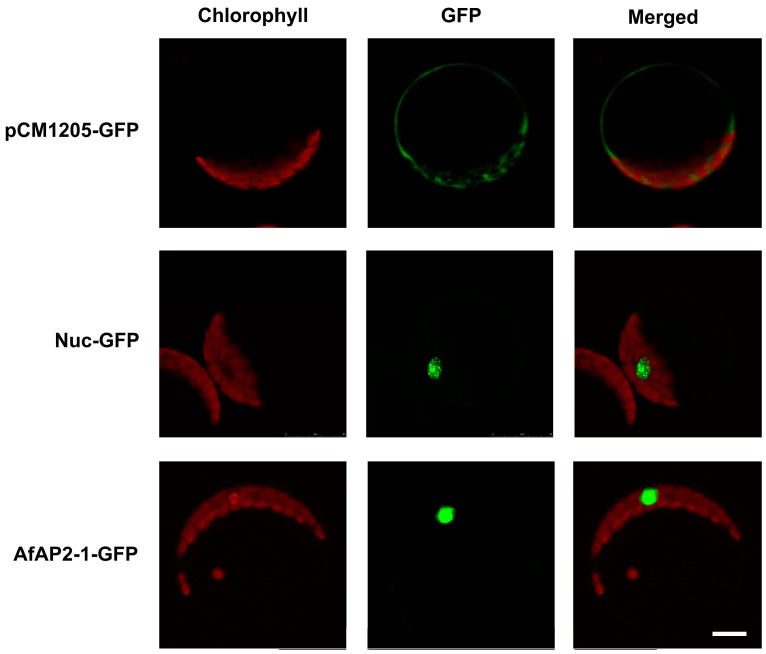
Subcellular localization of AfAP2-1-GFP fusion protein. Green fluorescence indicates GFP, and red fluorescence shows chloroplast auto-fluorescence. pCM1205-GFP: control lacking the transit peptide; Nuc-GFP: control with the nuclear localization signal of fibrillarin. AfAP2-1-GFP: GFP signals from the AfAP2-1-GFP fusion protein. The fluorescence signals were visualized using confocal laser scanning microscopy. Scale bar: 5 µm.

Based on a consensus sequence analysis, a completely conserved NLS was identified in the N terminus of AfAP2-1 protein (Figure 1A,B). To confirm the presumed nuclear localization of AfAP2-1, a fusion protein consisting of AfAP2-1 fused to the N terminus of green fluorescent protein (GFP) was transiently expressed in *Arabidopsis* protoplasts. As shown in Figure 5, the GFP protein was uniformly distributed throughout the cells, whereas the AfAP2-1-GFP fusion protein was localized predominantly in the nucleus, suggesting that the latter is constitutively localized to the nucleus.

### 2.7. Constitutive Overexpression of AfAP2-1 in Arabidopsis Causes Significantly Delayed Flowering Phenotype

**Figure 6:**
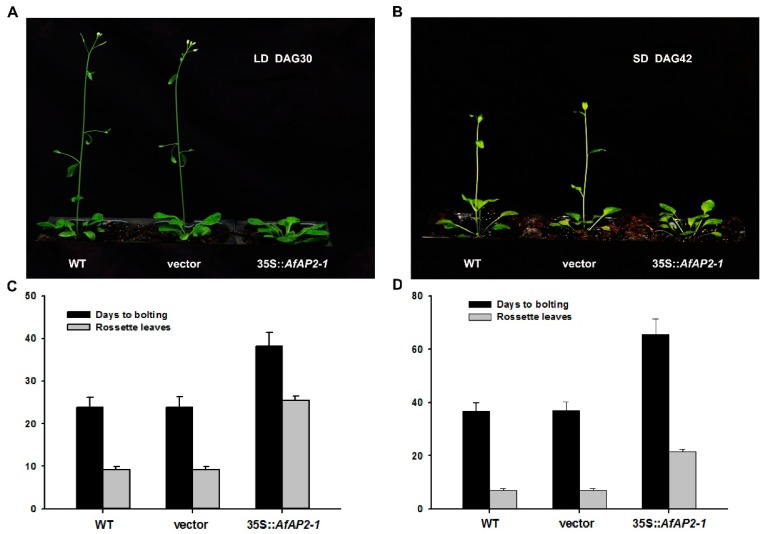
Heterologous expression of *AfAP2-1* in *Arabidopsis*. (**A**) Phenotypes of transgenic plants grown under LD conditions at 30 days after germination (DAG); (**B**) Phenotypes of transgenic plants grown under SD conditions at 42 DAG; (**C**) Days and rosette leaves to bolting of WT and transgenic plants grown under LD conditions; (**D**) Days and rosette leaves to bolting of WT and transgenic plants grown under SD conditions. Values are means ± standard deviation of the results from 26 individual plants of WT, 21 individual plants of WT transformed with the empty vector and 16 individual plants of *AfAP2-1*-OX lines.

To assess the involvement of AfAP2-1 in the regulation of flowering time, we overexpressed AfAP2-1 in WT and transformed the empty vector into the WT as a control. Notably, the bolting time did not differ between the WT and WT transformed with the empty vector, but the *AfAP2-1*-OX lines exhibited a significantly delayed flowering phenotype under both inductive LD and noninductive SD conditions (Figure 6A,B). *AfAP2-1*-OX lines bolted an average of 14.4 and 28.7 d later than the WT controls under LD and SD conditions, respectively. In addition, the *AfAP2-1*-OX lines exhibited 16.3 and 14.4 more rosette leaves than WT controls in average under LD and SD conditions, respectively (Figure 6C,D). These results indicated that the overexpression of *AfAP2-1* in *Arabidopsis* significantly delayed flowering time.

## 3. Discussion

The members of the APETALA2/Ethylene Responsive Element Binding Protein (AP2/EREBP) superfamily, which contain at least one AP2 domain, are responsive to multiple environmental stimuli and represent conservatively widespread TFs in the control of growth and developmental programs of the plants, protists, cyanobacteria and fungi [27,28]. The AP2/EREBP superfamily can be divided into the AP2 and EREBP families according to the number of AP2 domains. Specifically, members of this superfamily that are characterized by a tandem repeat of two AP2 domains are part of the AP2 family [28]. The AP2 family is further divided into two groups, AP2 and ANT, based on differences in the amino acid residues of the double AP2 domains and the NLS [28,29].

In this study, we provided the first report of the isolation of an *AP2*-like gene, *AfAP2-1*, from *A. fasciata*. A sequence analysis identified two conserved AP2 domains, a highly conserved putative NLS adjacent to AP2R1 and three other conserved motifs that have been identified in several AP2-like proteins (Figure 1B). These conserved domains and motifs may be essential for the functional conservation and evolution of AfAP2-1 [10,29,30,31] (Figure 1A,B).

### 3.1. Overexpression of AfAP2-1 in Arabidopsis Strongly Delayed Flowering under both LD and SD Conditions

Two copies of AP2 domains separated by a spacer region confer AP2 subfamily members with the ability to bind the promoter region of target genes [32]. Our results indicated that AfAP2-1 plays a role as a transactivator and that the activation domain is located in the C terminus, which did not contain two AP2 domains (Figure 4B). This result contradicted that obtained for AP2 isolated from *Brassica napus*, whose transcriptional activity was localized to the N terminus [33]. This difference indicates structural divergence during AP2 evolution.

TFs regulate the expression of numerous genes to mediate many patterning processes in the plant kingdom by binding to specific sites or motifs of DNA sequences [34,35]. The AP2/EREBP TF family, which contains nearly 7% of all TFs in *Arabidopsis*, are involved in multiple signalling processes [36]. Previous studies demonstrated that all six *AP2* family genes in *Arabidopsis* act redundantly to repress flowering [11,12,14,18], and at least TOE1 and SMZ can repress floral induction by inhibiting the photoperiod pathway [12,13]. To investigate the role of *AfAP2-1* in floral induction, we overexpressed this gene in *Arabidopsis*. Our results show that the heterogeneous overexpression of *AfAP2-1* in *Arabidopsis* significantly delayed flowering time under both the LD and SD conditions (Figure 6A,B). Furthermore, *AfAP2-1*-OX transgenic lines contained significantly more rosette leaves than WT plants (Figure 6C,D), indicating that *AfAP2-1* may play an important role in promoting the vegetative growth of *Arabidopsis*. TOE1 can bind to an AT-rich element in the *FT* promoter near the CONSTANS (CO)-binding site to convey a photoperiodic signal to regulate flowering time [13]. Our study identified several AT-rich elements in the promoter of *AfFT2*, a putative florigen in *A. fasciata*, implying the putative direct binding site(s) of members of the AP2 subfamily, such as AfAP2-1 (data not shown). Furthermore, the relative expression of *AfAP2-1* was under the control of the circadian rhythm (Figure 3C), similar to the control of *TOE1*, *TOE2*, *SMZ* and *SNZ* [13]. This regulation implies that AfAP2-1 proteins may function not only in the morning and afternoon but also late at night (Figure 3C). Further investigation should be focused on verifying the interactions between AfAP2-1 and *FT*-like genes or other flowering integrators in *A. fasciata*.

### 3.2. The Abundance of AfAP2-1 Transcripts Is Negatively Regulated by Exogenous Ethylene Treatment

Although ethylene is widely used to induce the flowering of bromeliads to both avoid the desynchrony of natural flowering and reduce time and manpower costs, this forced flowering also depends on the plant development age, *i.e.*, adult plants can successfully bolt, whereas juvenile plants cannot. To date, the precise mechanism by which ethylene induces flowering is unknown. Our previous study identified several members of the AP2/EREBP family that may be involved in both the ethylene-responsive and age-dependent flowering pathways [37], indicating that *AP2*-like genes may play a role in this complex process. Jung [11] demonstrated that the transcript levels of *TOE1* and *TOE2* gradually decreased throughout plant growth. *CfTOE1* is also involved in age-dependent flowering in *Cardamine flexuosa* [38]. In our study, the expression of *AfAP2-1* also depended on the plant development age; it was relatively high in juvenile plants, decreased in adult plants prior to flower bud differentiation, and further decreased in 39-DAF adult plants (Figure 3A). To date, the correlation between ethylene and AP2 subfamily members has not yet been investigated. Here, we showed that the transcripts levels of *AfAP2-1* in all detected organs dramatically decreased after treatment with 400 μL·L^−1^ of ethrel for 1 h and were almost undetectable after 6 h, indicating the rapid response of *AfAP2-1* to exogenous ethylene treatment both in juvenile and in adult plants (Figure 3D). Exogenous ethylene treatment after 6 h also strongly induced *AfFT2* expression in central leaves and stems (unpublished data). The response time delay of *AfFT2* compared with that of *AfAP2-1* implied that *AfAP2-1* may be upstream of *AfFT2*. These results all indicate that a decrease in the expression of *AfAP2-1* below a threshold may induce bolting in adult bromeliads treated with exogenous ethylene, but not juveniles, or that some other regulators may be involved in this complex process. Further studies should focus on verifying the precise mechanism by which ethylene affects *AfAP2-1*.

In conclusion, our results indicate that *AfAP2-1* is an AP2 family gene expressed in *A. fasciata*. The overexpression of *AfAP2-1* in *Arabidopsis* significantly delayed flowering, implying that the molecular mechanism by which *AfAP2-1* affects flowering may be partially conserved between *Arabidopsis* and *A. fasciata*. Further analyses of the interactions between ethylene-responsive factors and *AfAP2-1* and transgenic plants of *A. fasciata* will help us to better understand the function of *AfAP2-1* in flowering and provide a theoretical basis for the genetic engineering of *A. fasciata* and possibly other species of bromeliads.

## 4. Materials and Methods

### 4.1. Plant Materials and Growth Conditions

The juvenile (6–8 months) and adult (11–14 months) plants of *A. fasciata* used in this study were grown in a greenhouse (ambient temperature was 30–32 °C) located in the experimental area at the Institute of Tropical Crop Genetic Resources of the Chinese Academy of Tropical Agricultural Sciences (CATAS). The mature leaves, central leaves, stems, roots and variable organs of flowers were collected and immediately frozen in liquid nitrogen for further research.

The seeds of both the wild-type and transgenic lines of *Arabidopsis* were incubated in the dark at 4 °C for 2 days to ensure synchronized germination and were then surface-sterilized before being plated on Murashige and Skoog (MS)-supplemented medium containing 2% sucrose and 0.8% agar. The plates were grown in a chamber under LD (16 h light and 8 h dark) or SD (8 h light and 16 h dark) conditions with a photon flux density of 100 μmol·m^−2^·s^−1^ at 23 °C. Ten days later, the seedlings were transplanted into turf soil for continuous growth.

### 4.2. Isolation and Sequencing of the AfAP2-1 Gene

To isolate the *AfAP2-1* gene, total RNA was extracted from the central leaves of *A. fasciata* using Hexadecyl trimethyl ammonium bromide (CTAB) methods [39]. Based on the transcriptome data of *A. fasciata*, 5′ and 3′ RACE was performed using a SMARTer™ RACE cDNA Amplification Kit (Clontech, Tokyo, Japan) according to the manufacturer’s instructions. The primers used here are listed in Table S1. The specific 5′ and 3′ fragments were cloned into pEASY-blunt vectors (Transgene, Beijing, China) and sequenced by Thermo Fisher Scientific (Guangzhou, China).

### 4.3. Bioinformatic Analysis

The molecular weight and pI of the deduced amino acid sequence of AfAP2-1 were calculated online using the ProtParam tool [40]. The ORF was predicted using ORF Finder [41]. The homologous protein sequences of AfAP2-1 were identified using BLAST [42]. Amino acid sequence alignment was carried out using Cluster Omega [43]. A phylogenetic tree was constructed with MEGA version 6 [44] using the neighbour-joining method with 1000 bootstrap replicates.

### 4.4. qRT-PCR Analysis

Total RNA was extracted from various organs of *A. fasciata* using CTAB methods [39] or from the leaves of *Arabidopsis* using an E.Z.N.A. Total RNA Kit I (Omega, Norcross, UK). First-strand cDNA was synthesized according to the manufacturer’s instructions for the TransScript One-Step gDNA Removal and cDNA Synthesis SuperMix (Transgen, Beijing, China). qRT-PCR was performed using TransStart Tip Green qPCR SuperMix (Transgen) on a Therma PikoReal 96™ Real-Time PCR System (Thermo Fisher Scientific, New York, NY, USA). Two biological replicates and three technical replicates for each sample were included in each PCR assay. The data were analysed using the 2^−ΔΔ*C*t^ method, as described by Livak and Schmittgen [45]. The *α-actin* and *β-tubulin* genes of *A. fasciata* were used as the internal control. All primers used for qRT-PCR are listed in Table S1.

### 4.5. Plant Expression Vector Construction and Arabidopsis Transformation

To construct the overexpression vector 35S::*AfAP2-1*, the coding sequence (CDS) of *AfAP2-1* was excised from the pEASY-blunt cloning vector using the restriction enzymes KpnI and XbaI, after which the fragment was cloned into pBI121.

The confirmed construct was transformed into Col-0 background *Arabidopsis* using agrobacterium strain EHA105, as described by Clough and Bent [46]. Transgenic seedlings were selected on MS agar medium supplemented with 25 mg/L hygromycin and 50 mg/L kanamycin and verified by genomic PCR and RT-PCR. The T2 transgenic homologous lines were selected for further analyses.

### 4.6. Transactivation Analysis of AfAP2-1 in Yeast Cells

The ORF (1–453 amino acids), the AP2 domains containing the N terminus (1–294 amino acids) and the C terminus (284–453 amino acids) were fused in frame with the yeast GAL4 DNA binding domain vector pGBKT7 (pBD) to yield the expression vectors pBD-AfAP2-1, pBD-AfAP2-1N and pBD-AfAP2-1C. pBD and pGAL4 were used as negative and positive controls, respectively. The primers used are listed in Table S1. The constructs were transformed into yeast strain Y2HGold containing the reporter genes *LacZ* and *AbA* according to the instructions in the Matchmaker^TM^ Gold Yeast Two-Hybrid System User Manual (Clontech, Tokyo, Japan). Putative positive yeast colonies were selected on synthetic dropout (SD) medium for tryptophan (Clontech, Tokyo, Japan). After confirmation by PCR, the positive yeast transformants were scratched, and 2 μL of diluted liquid strains was dripped onto SD-deleting tryptophan medium containing X-α-gal and AbA. The cultures were then incubated at 30 °C for 3 days to determine the transactivation activity.

### 4.7. Subcellular Localization of AfAP2-1 Protein

The full-length CDS of *AfAP2-1* was amplified using the primers AfAP2-1-GFP-F and AfAP2-1-GFP-R (Table S1) to generate the 35S::AfAP2-1-GFP. The resulting fusion constructs and empty vector 35S::GFP were then transformed into the protoplasts of *Arabidopsis*. GFP fluorescence was observed by confocal scanning microscopy (OLYMPUS FV1000, OLYMPUS, Tokyo, Japan). For GFP, 488 and 505–530 nm were used for excitation and emission, respectively. For chlorophyll auto-fluorescence, a 488 nm laser line and a 650–750 nm bandpass filter were used for excitation and emission, respectively.

### 4.8. Exogenous Ethylene Treatment of A. fasciata

To test the response to ethylene, juvenile (6–8 months) and adult (11–14 months) plants of *A. fasciata* grown in a greenhouse (ambient temperature of 30–32 °C) were treated with 400 μL·L^−1^ of ethrel for 1, 6 or 24 h. The mature leaves, central leaves, stems and roots were then physically isolated and immediately frozen in liquid nitrogen. Total RNA was extracted as described by Cong *et al.* [39]. First-strand cDNA was synthesized using TransScript One-Step gDNA Removal and cDNA Synthesis SuperMix (Transgen) for further research.

### 4.9. Data Analysis

All data were represented as the mean ± standard deviation of three or more biological replicates.

## Supplementary Materials

Supplementary materials can be found at http://www.mdpi.com/1422-0067/17/3/303/s1.

## Abbreviations

*AP2*: *APETALA2*; TF: transcription factor; *TOE1*: *TARGET OF EAT 1*; *SMZ*: *SCHLAFMÜTZE;* *SNZ*: *SCHNARCHZAPFEN;* miR172: microRNA172; *FT*: *Flowering Locus T*; GA: gibberellin acid; EIN3: Ethylene Insensitive 3; CTR1: CONSTITUTIVE TRIPLE RESPONSE1; *LFY*: *LEAFY*; *SOC1*: *SUPPRESSOR OF OVEREXPRESSION OF CONSTANS 1*; AVG: Aviglycine; ACC synthase: 1-amino-cyclopropane-1-carboxylate synthase; Col-0: Columbia-0; SD: short-day; LD: long-day; RACE: rapid-amplification of cDNA ends; DAF: day-after-flowering; GFP: green fluorescent protein; DAG: days after germination; AP2/EREBP: APETALA2/Ethylene Responsive Element Binding Protein.
