# Supplementary material for: AfAP2-1, An Age-Dependent Gene of Aechmea fasciata, Responds to Exogenous Ethylene Treatment
Source: Int J Mol Sci. 2016 Feb 27;17(3):303. doi: 10.3390/ijms17030303 (PMC4813167; doi:10.3390/ijms17030303)
Supplement: Supplementary file 1 [file ijms-17-00303-s001.docx]

*AfAP2-1*, An Age-Dependent Gene of *Aechmea fasciata*, Responds to Exogenous Ethylene Treatment

Ming Lei, Zhi-Ying Li, Jia-Bin Wang, Yun-Liu Fu, Meng-Fei Ao and Li Xu

**Table S1.** Primers used in this study.

| **Name** | **Sequences (From 5′ to 3′)** |
| --- | --- |
| *AfAP2-1* 5′ RACE GSP1 | AAC TTG TTT CCC GCA GTC CC |
| *AfAP2-1* 5′ RACE GSP2 | ATG TGC GAC TCC CAT CTA CC |
| *AfAP2-1* 3′ RACE GSP1 | CCA AGC AGT TAT TTC CCC TCA |
| *AfAP2-1* 3′ RACE GSP2 | TCG TCG TCT AAT AGG TCG CA |
| *AfAP2-1*-OX F | CTA GTC GAT ATC AGC TCT TGA AGA AAT TCA AAG |
| *AfAP2-1*-OX R | CGG GGT ACC ATG ATG CTC GAT CTG AAC ATG GCC G |
| AfAP2-1-pGBKT7 F | CCG GAA TTC ATG CTG GAT CTA AAC GTG TCC |
| AfAP2-1-pGBKT7 R | ACG CGT CGA CTT AGC TCT TGA AAG AGT AAC CGG |
| AfAP2-1N-pGBKT7 F | CCG GAA TTC ATG CTG GAT CTA AAC GTG TCC |
| AfAP2-1N-pGBKT7 R | ACG CGT CGA CTT AGC TAA ACA GTT CGC CTT C |
| AfAP2-1C*-*pGBKT7 F | CCG GAA TTC CCC AAC ACA TAT GAA GGC GAA C |
| AfAP2-1C-pGBKT7 R | ACG CGT CGA CTT AGC TCT TGA AAG AGT AAC CGG |
| AfAP2-1-GFP F | GCT CTA GAA TGC TGG ATC TAA ACG TGT CCG |
| AfAP2-1-GFP R | CGG AAT TCG CTC TTG AAA GAG TAA CCG GAT |
| *AfAP2-1* qRT-PCR F | GTG GCT TCG TCC TCG TCA TC |
| *AfAP2-1* qRT-PCR R | GCA GTG TCA AAT CCT CCC AAA TA |
| *Afα-actin* qRT-PCR F | GAG CAG CAT GAA GAT CAA GG |
| *Afα-actin* qRT-PCR R | CAT CTG CTG GAA AGT GCT GA |
| *Afβ-tubulin* qRT-PCR F | TCC AGA CCA ACC TGG TCC CTT AT |
| *Afβ-tubulin* qRT-PCR R | ACA TCC TTG GGG ACA ACA TCA CC |
